# Diabetes Distress Among US Adults With Diagnosed Diabetes, 2021

**DOI:** 10.5888/pcd22.240287

**Published:** 2025-02-24

**Authors:** Dayna S. Alexander, Ryan Saelee, Betsy Rodriguez, Alain K. Koyama, Yiling J. Cheng, Shichao Tang, Rachel E. Rutkowski, Kai McKeever Bullard

**Affiliations:** 1 Division of Diabetes Translation, National Center for Chronic Disease Prevention and Health Promotion, Centers for Disease Control and Prevention, Atlanta, Georgia

## Abstract

National prevalence of diabetes distress is unknown among US adults. This cross-sectional study examined the prevalence among US adults with diabetes using 2021 National Health Interview Survey data. Multivariable multinomial logistic regressions were used to estimate adjusted prevalence and prevalence ratios for diabetes distress. Adjusted prevalence of moderate and severe diabetes distress was 24.3% (95% CI, 22.5%–26.1%) and 6.6% (95% CI, 5.6%–7.8%), respectively. Prevalence was higher among people aged 18 to 64 years, women, and those with lower incomes. Findings highlight the importance of examining economic and social factors and integrating diabetes distress screening into diabetes management and services.

**Summary**

**What is already known on this topic?**

Diabetes distress can negatively affect diabetes care and management.

**What is added by this report?**

In 2021, more than half of US adults with diabetes had diabetes distress, including 7% with severe diabetes distress and 24% with moderate diabetes distress. Diabetes distress was higher among people aged 18 to 64 years, women, and those with lower income.

**What are the implications for public health practice?**

Researchers can assess the prevalence of diabetes distress and examine economic and social factors that contribute to differences. Interventions including diabetes distress screening, behavioral therapy (such as stress management and psychoeducation), and family support may improve diabetes management and services.

## Objective

Diabetes prevalence has increased among US adults aged 18 years or older, and in 2021, 29.7 million people were diagnosed with the disease (1). People living with diabetes are more likely to experience adverse mental, social, and physical health effects that result in diabetes distress (DD). DD refers to the emotional and psychological difficulty among people with diabetes when they manage their condition (2). Approximately 18% to 40% of people with diabetes experience significant DD, with 18-month cumulative incidence ranging from 38% to 48% (2). DD is associated with lower glycemic control, decreased self-glucose monitoring, and poor medication management (3). Study findings have illustrated that people with high self-efficacy (ie, a person’s confidence in their ability to achieve a goal) have lower DD compared with those with low self-efficacy (4). Thus, prior findings (3,4) demonstrate the importance of assessing DD among people living with diabetes to support behavioral change by implementing multilevel, culturally tailored interventions. We aimed to examine the prevalence and associated distress factors — including sociodemographic, treatment, and health status — among US adults diagnosed with diabetes by using National Health Interview Survey (NHIS) data (5).

## Research Design and Methods

This cross-sectional analysis used self-reported data from the 2021 NHIS, a national representative survey of the US civilian noninstitutionalized population conducted by the Centers for Disease Control and Prevention’s National Center for Health Statistics (5). The 2021 NHIS introduced 2 supplemental questions based on a modified version of a question on the Diabetes Distress Scale that assesses whether someone is “feeling overwhelmed by the demands of living with diabetes” (6). Respondents aged 18 years or older self-reported diagnosed diabetes based on the question, “Have you ever been told by a doctor or health professional (other than during pregnancy, if female) that you have diabetes?” We used the question, “During the past month, how often have you felt overwhelmed by the demands of living with diabetes? Would you say always, usually, sometimes, rarely, or never?” to classify DD as severe (always), moderate (usually or sometimes), mild (rarely), and none (never). We also assessed how overwhelmed respondents were currently compared with before the COVID-19 pandemic, by DD level. Covariates included age, sex, race and ethnicity, education, imputed family poverty-to-income ratio (PIR; variable RATCAT_A), living alone, cost-related medication/insulin underuse, diabetes duration, self-reported health, diagnosed depression, and diagnosed anxiety. The analytic sample included 3,096 respondents, excluding those with missing data (n = 38).

To calculate estimates representing the US population with diagnosed diabetes and accounting for the complex design and weights of the NHIS, we used SAS-callable SUDAAN version 11.0.3 (Research Triangle Institute). We compared differences in characteristic distributions by DD level using Pearson χ^2^ tests. Multivariable multinomial logistic regressions with predictive margins were used to estimate adjusted prevalence and prevalence ratios with 95% CIs for DD by subgroup, adjusting for covariates. All estimates met National Center for Health Statistics data presentation standards for proportions (7). Significance was evaluated using *P* < .05 Pearson or 95% CI (prevalence, prevalence ratios).

## Results

Among US adults with diabetes, an estimated 1.6 million (6.6%) had severe DD, 5.8 million (24.3%) had moderate DD, 4.8 million (19.9%) had mild DD, and 11.8 million (49.3%) had no DD (Table 1). Characteristics of adults with diabetes varied by level of DD. Specifically, age, sex, race and ethnicity, PIR, cost-related medication/insulin underuse, self-reported health, diagnosed depression, and diagnosed anxiety were significantly associated with DD (*P* < .05). Compared with their counterparts, adjusted prevalence of severe DD was higher in adults aged 18 to 49 and 50 to 64 years, Hispanic and non-Hispanic Black adults, adults with a PIR of less than 3.00, adults who reported cost-related insulin underuse, adults with fair/poor self-reported health, and adults with diagnosed depression or anxiety (Table 2). We observed similar but attenuated patterns for adjusted prevalence of moderate DD, except that estimates were also higher among women and not significantly different across race and ethnicity groups. In contrast, adjusted prevalence of mild DD was similar among most subgroups, apart from lower prevalence among adults with less than high school education and those with the lowest income compared with their counterparts. The adjusted percentages of adults reporting no DD was higher among those aged 65 years or older, male respondents, those with a PIR of 3.00 or higher (compared with those with a PIR of 1.00–2.99), those who did not report cost-related medication/insulin underuse, those diagnosed with diabetes of less than 15 years, those with excellent/very good/good self-reported health, and those with no diagnosed depression or anxiety (Table 2). Compared with 3.6% (95% CI, 2.6%–5.0%) of US adults with diabetes without DD, 37.6% (95% CI, 29.8%–46.2%) of those with severe DD, 25.2% (95% CI, 21.6%–29.2%) of those with moderate DD, and 9.4% (95% CI, 7.0%–12.5%) of those with mild DD reported being more overwhelmed living with diabetes now than before the COVID-19 pandemic (Figure).

**Table 1:** Characteristics of US Adults With Diabetes (N = 3,096), by Level of Diabetes Distress, National Health Interview Survey, 2021^a^

Characteristic	Severe diabetes distress	Moderate diabetes distress	Mild diabetes distress	No diabetes distress	*P* value^b^
Unweighted no.	200	723	634	1,539	—
Represented population size, no. in millions (%)	1.6 (6.6)	5.8 (24.3)	4.8 (19.9)	11.8 (49.3)	—
**Age, y**					
18–49	23.6 (3.7)	26.6 (2.1)	14.1 (1.9)	13.9 (1.1)	<.001
50–64	48.5 (4.2)	37.7 (2.2)	37.3 (2.4)	36.4 (1.6)	
≥65	28.0 (3.4)	35.7 (2.0)	48.6 (2.4)	49.7 (1.6)	
**Mean age, y**	57.6 (1.0)	58.2 (0.7)	62.8 (0.7)	63.4 (0.5)	<.001
**Sex**					
Female	50.9 (4.1)	56.6 (2.2)	50.2 (2.3)	43.1 (1.5)	<.001
Male	49.1 (4.1)	43.4 (2.2)	49.8 (2.3)	56.9 (1.5)	
**Race and ethnicity**					
Hispanic	30.2 (4.5)	22.0 (2.1)	16.7 (2.1)	16.5 (1.4)	<.001
NH Black	23.0 (3.6)	17.3 (1.7)	13.4 (1.7)	15.3 (1.2)	
NH White	37.2 (4.1)	49.6 (2.4)	62.5 (2.4)	60.0 (1.7)	
NH Other	9.7 (2.8)	11.1 (1.7)	7.5 (1.3)	8.2 (0.9)	
**Education**					
Less than high school	27.5 (4.2)	18.1 (1.6)	13.0 (1.7)	17.0 (1.2)	.01
High school/GED	36.0 (4.2)	34.1 (2.3)	34.4 (2.4)	32.3 (1.4)	
Some college or higher	36.5 (4.1)	47.8 (2.2)	52.6 (2.4)	50.7 (1.5)	
**Family poverty-to-income ratio**					
<1.00	25.9 (3.7)	14.3 (1.3)	9.5 (1.5)	12.0 (1.1)	<.001
1.00–2.99	51.7 (4.2)	47.2 (2.2)	44.9 (2.4)	39.3 (1.5)	
≥3.00	22.4 (3.3)	38.5 (2.1)	45.6 (2.4)	48.6 (1.5)	
**Living alone**					
Yes	24.3 (3.0)	19.1 (1.4)	21.9 (1.5)	22.2 (1.0)	.25
No	75.7 (3.0)	80.9 (1.4)	78.2 (1.5)	77.8 (1.0)	
**Cost-related medication/insulin underuse^c^**					
Yes	31.1 (3.8)	23.8 (1.8)	13.7 (1.8)	9.1 (1.0)	<.001
No	69.0 (3.8)	76.2 (1.8)	86.3 (1.8)	91.0 (1.0)	
**Duration of diabetes, y**					
<15	50.4 (4.1)	57.8 (2.2)	59.6 (2.2)	61.5 (1.5)	.05
≥15	47.0 (4.0)	39.3 (2.1)	38.2 (2.2)	34.9 (1.4)	
**Self-reported health**					
Excellent/very good/good	28.1 (3.8)	48.2 (2.2)	64.1 (2.3)	71.6 (1.4)	<.001
Fair/poor	71.9 (3.8)	51.8 (2.2)	35.9 (2.3)	28.4 (1.4)	
**Depression diagnosis**					
Yes	50.7 (4.2)	34.2 (2.0)	23.4 (1.9)	17.1 (1.3)	<.001
No	49.3 (4.2)	65.8 (2.0)	76.6 (1.9)	82.9 (1.3)	
**Anxiety diagnosis**					
Yes	50.3 (4.0)	26.6 (1.9)	19.9 (1.9)	13.7 (1.1)	<.001
No	49.7 (4.0)	73.4 (1.9)	80.1 (1.9)	86.3 (1.1)	

Abbreviations: —, not applicable; GED, general educational development; NH, non-Hispanic.

a Estimates are weighted percentage (SE) unless otherwise noted.

b Pearson χ^2^ tests were performed to assess whether differences existed in the distribution of characteristics by diabetes distress level.

c Based on a positive response to survey questions asking about having to take less medication/insulin, delay in getting medication/insulin, or skipping medication/insulin to save money.

**Table 2:** Adjusted Prevalence and Prevalence Ratios of Diabetes Distress Among US Adults With Diabetes (N = 3,096), National Health Interview Survey, 2021^a^

Characteristic	Severe diabetes distress		Moderate diabetes distress		Mild diabetes distress		No diabetes distress	
	% (95% CI)	aPR (95% CI)	% (95% CI)	aPR (95% CI)	% (95% CI)	aPR (95% CI)	% (95% CI)	aPR (95% CI)
**Overall**	6.6 (5.6–7.8)	—	24.3 (22.5–26.1)	—	19.8 (18.2–21.5)	—	49.3 (47.2–51.5)	—
**Age, y**								
18–49	8.0 (5.7–11.3)	1.89 (1.20–2.97)^b^	35.5 (30.2–41.2)	1.83 (1.50–2.24)^b^	16.4 (12.6–21.0)	0.76 (0.58–1.01)	40.1 (34.7–45.7)	0.73 (0.63–0.84)^b^
50–64	8.6 (6.7–11.0)	2.03 (1.44–2.87)^b^	24.8 (22.0–27.9)	1.28 (1.07–1.53)^b^	19.7 (17.1–22.7)	0.92 (0.77–1.11)	46.8 (43.6–50.1)	0.85 (0.78–0.93)^b^
≥65	4.2 (3.2–5.5)	1 [Ref]	19.4 (17.0–22.0)	1 [Ref]	21.4 (19.0–24.0)	1 [Ref]	55.0 (51.9–58.1)	1 [Ref]
**Sex**								
Female	6.5 (5.3–8.0)	0.97 (0.71–1.31)	27.9 (25.5–30.5)	1.34 (1.16–1.56)^b^	20.8 (18.7–23.2)	1.10 (0.94–1.29)	44.8 (41.9–47.6)	0.84 (0.77–0.91)^b^
Male	6.7 (5.3–8.5)	1 [Ref]	20.8 (18.5–23.4)	1 [Ref]	18.9 (16.7–21.4)	1 [Ref]	53.5 (50.5–56.6)	1 [Ref]
**Race and ethnicity**								
Hispanic	8.6 (6.1–12.1)	1.75 (1.13–2.69)^b^	26.0 (21.4–31.1)	1.14 (0.92–1.42)	18.4 (14.3–23.4)	0.85 (0.65–1.11)	47.0 (41.4–52.6)	0.93 (0.81–1.06)
NH Black	8.5 (6.2–11.6)	1.73 (1.15–2.58)^b^	24.6 (20.6–29.0)	1.08 (0.88–1.33)	17.0 (13.4–21.4)	0.78 (0.61–1.00)	49.9 (44.6–55.1)	0.99 (0.88–1.11)
NH White	4.9 (3.9–6.3)	1 [Ref]	22.8 (20.5–25.2)	1 [Ref]	21.7 (19.6–24.0)	1 [Ref]	50.6 (47.8–53.4)	1 [Ref]
**Education**								
Less than high school	7.8 (5.3–11.2)	1.35 (0.82–2.20)	23.7 (19.4–28.5)	0.96 (0.77–1.21)	14.7 (11.3–18.9)	0.69 (0.52–0.92)^b^	53.9 (48.5–59.2)	1.11 (0.99–1.25)
High school/GED	6.9 (5.2–8.9)	1.19 (0.81–1.74)	24.4 (21.1–27.9)	0.99 (0.83–1.19)	20.3 (17.4–23.6)	0.96 (0.79–1.16)	48.5 (44.7–52.3)	1.00 (0.91–1.10)
Some college or higher	5.8 (4.4–7.6)	1 [Ref]	24.5 (22.0–27.3)	1 [Ref]	21.2 (18.9–23.7)	1 [Ref]	48.5 (45.6–51.4)	1 [Ref]
**Family poverty-to-income ratio**								
<1.00	11.2 (8.1–15.2)	3.06 (1.93–4.86)^b^	24.3 (20.0–29.2)	1.09 (0.87–1.37)	14.4 (10.9–18.8)	0.70 (0.52–0.95)^b^	50.1 (44.4–55.8)	0.93 (0.82–1.06)
1.00–2.99	7.8 (6.1–9.8)	2.13 (1.45–3.12)^b^	26.5 (23.8–29.4)	1.19 (1.02–1.40)^b^	20.7 (18.2–23.5)	1.01 (0.84–1.21)	45.0 (41.9–48.3)	0.84 (0.77–0.92)^b^
≥3.00	3.7 (2.7–5.0)	Ref	22.2 (19.7–25.0)	1 [Ref]	20.6 (18.1–23.2)	1 [Ref]	53.6 (50.3–56.8)	1 [Ref]
**Living alone**								
Yes	7.5 (5.8–9.6)	1.18 (0.86–1.61)	22.4 (19.6–25.6)	0.90 (0.77–1.07)	19.6 (17.2–22.3)	0.99 (0.84–1.16)	50.5 (46.9–54.0)	1.03 (0.94–1.12)
No	6.4 (5.2–7.8)	1 [Ref]	24.8 (22.7–27.0)	1 [Ref]	19.9 (18.0–21.9)	1 [Ref]	49.0 (46.5–51.6)	1 [Ref]
**Cost-related medication/insulin underuse^c^**								
Yes	11.8 (8.8–15.5)	2.12 (1.51–2.98)^b^	35.6 (30.4–41.3)	1.60 (1.34–1.91)^b^	19.5 (15.4–24.5)	0.98 (0.75–1.27)	33.1 (27.7–39.0)	0.63 (0.53–0.76)^b^
No	5.6 (4.5–6.8)	1 [Ref]	22.3 (20.4–24.2)	1 [Ref]	20.0 (18.2–22.0)	1 [Ref]	52.2 (49.8–54.6)	1 [Ref]
**Duration of diabetes, y**								
<15	5.3 (4.1–6.7)	0.59 (0.42–0.82)^b^	22.6 (20.4–25.0)	0.84 (0.72–0.98)^b^	20.2 (18.2–22.4)	1.02 (0.86–1.21)	51.9 (49.1–54.7)	1.17 (1.07–1.28)^b^
≥15	9.0 (7.2–11.1)	1 [Ref]	27.0 (24.0–30.3)	1 [Ref]	19.8 (17.2–22.6)	1 [Ref]	44.3 (41.1–47.5)	1 [Ref]
**Self-reported health**								
Excellent/very good/good	3.2 (2.4–4.4)	1 [Ref]	19.5 (17.5–21.8)	1 [Ref]	20.6 (18.5–22.9)	1 [Ref]	56.6 (53.8–59.4)	1 [Ref]
Fair/poor	11.2 (9.2–13.7)	3.44 (2.35–5.03)^b^	32.0 (28.8–35.3)	1.64 (1.41–1.92)^b^	18.8 (16.3–21.7)	0.93 (0.77–1.11)	38.0 (34.8–41.2)	0.66 (0.60–0.73)^b^
**Depression diagnosis**								
Yes	12.9 (10.2–16.1)	2.91 (2.10–4.02)^b^	31.5 (27.9–35.3)	1.44 (1.24–1.68)^b^	18.6 (15.6–22.0)	0.92 (0.75–1.11)	37.0 (32.9–41.3)	0.69 (0.61–0.79)^b^
No	4.4 (3.5–5.6)	1 [Ref]	21.9 (19.8–24.0)	1 [Ref]	20.3 (18.5–22.4)	1 [Ref]	53.4 (50.8–56.0)	1 [Ref]
**Anxiety diagnosis**								
Yes	15.3 (12.1–19.2)	3.63 (2.69–4.89)^b^	28.5 (24.6–32.6)	1.23 (1.04–1.45)^b^	19.9 (16.4–23.9)	1.00 (0.81–1.23)	36.3 (31.9–41.0)	0.69 (0.60–0.79)^b^
No	4.2 (3.4–5.2)	1 [Ref]	23.2 (21.3–25.3)	1 [Ref]	19.9 (18.1–21.9)	1 [Ref]	52.6 (50.2–55.0)	1 [Ref]

Abbreviations: aPR, adjusted prevalence ratio; GED, general educational diploma; NH, non-Hispanic.

a Estimates are adjusted prevalences and prevalence ratios with their respective 95% CI calculated from multinomial logistic regression adjusted for continuous age, sex, race and ethnicity, and continuous family poverty to income ratio.

b Significant at *P* < .05.

c Based on a positive response to survey questions asking about having to take less medication/insulin, delay in getting medication/insulin, or skipping medication/insulin to save money.

**Figure:**
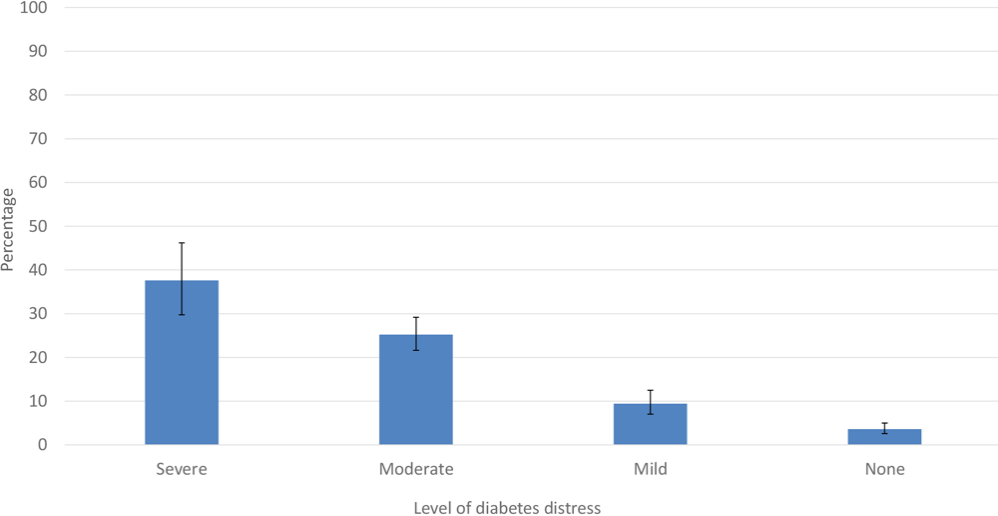
Percentage of US adults with diabetes who reported currently feeling more overwhelmed than before the COVID-19 pandemic. Responses based on the survey question, “Compared with the time before the coronavirus pandemic, would you say that you now feel more overwhelmed by the demands of living with diabetes, less overwhelmed, or about the same as before the pandemic?”

**Table:** 

Level of diabetes distress	US adults who report feeling more overwhelmed than before COVID-19, % (95% CI)
Severe	37.6 (29.8–46.2)
Moderate	25.2 (21.6–29.2)
Mild	9.4 (7.0–12.5)
None	3.6 (2.6–5.0)

## Discussion

We found that among US adults with diagnosed diabetes, 12.2 million (half of those with diabetes) are estimated to have severe, mild, or moderate DD. Our findings are consistent with previously reported point estimates of DD among US adults ranging from moderate to severe in various settings (8). We found that women were less likely than men to have no DD, showing that sex is a major demographic factor associated with DD (9). Although the cause is unknown, different coping strategies and stress management among the sexes may play a role in diabetes distress. Whereas Gahlan et al (10) found that lower level of education was associated with DD, we did not observe a significant association of educational attainment and DD. Our findings demonstrated that adults aged 65 years or older were less likely to have severe DD. This finding was consistent with prior research that postulated that older adults with type 2 diabetes experience DD but that they practice emotional regulation strategies (eg, reappraisal) (11).

This study is subject to limitations. First, results were based on a single-item definition of DD limited to the past month, which may misclassify some individuals; however, our estimates are similar to other studies in various populations and settings. Second, we did not have information on duration of DD, only on perceived severity of DD. Third, small sample sizes limited reliable estimation of DD prevalence among certain subgroups, such as by disaggregated race and ethnicity and by diabetes type.

This study provides the first national estimates of DD prevalence and highlights the importance of associated factors, such as sex, income, age, and race and ethnicity. Continued investment in DD data collection may be warranted to monitor changes in DD over time and examine additional economic and social factors contributing to DD-related disparities. Assessing the differences and impact of DD by diabetes type to guide individualized and population-level interventions is also needed. Program interventions integrating DD screening, behavioral therapy (eg, stress management, psychoeducation [ie, cognitive–behavioral, individual, and group-based therapy]), and family support (12) may improve diabetes management and services.
